# LASS2 suppresses metastasis in multiple cancers by regulating the ferroptosis signalling pathway through interaction with TFRC

**DOI:** 10.1186/s12935-024-03275-8

**Published:** 2024-02-28

**Authors:** Yunfei Huang, Jie Du, Dan Li, Wei He, Zhouheng Liu, Li Liu, Xiaoli Yang, Xiaoming Cheng, Rui Chen, Yan Yang

**Affiliations:** 1 Department of Laboratory Medicine, Affiliated Hospital of Zunyi Medical University, 149 Dalian Road, Zunyi, 563000 Guizhou China; 2 School of Laboratory Medicine, Zunyi Medical University, Zunyi, 563000 Guizhou China; 3 Department of General Surgery, Affiliated Hospital of Zunyi Medical University, Zunyi, 563000 Guizhou China; 4 Department of General Surgery, The Second Affiliated Hospital of Zunyi Medical University, Zunyi, 563000 Guizhou China; 5 School of Forensic Medicine, Zunyi Medical University, Zunyi, 563000 Guizhou China

**Keywords:** LASS2, TFRC, Ferroptosis, Multiple cancers, Metastasis

## Abstract

**Background:**

As a key enzyme in ceramide synthesis, longevity assurance homologue 2 (LASS2) has been indicated to act as a tumour suppressor in a variety of cancers. Ferroptosis is involved in a variety of tumour processes; however, the role of LASS2 in regulating ferroptosis has yet to be explored. This article explores the potential underlying mechanisms involved.

**Methods:**

Bioinformatics tools and immunohistochemical staining were used to evaluate LASS2 expression, and the results were analysed in relation to overall survival and clinical association in multiple cancers. Coimmunoprecipitation-coupled liquid chromatography-mass spectrometry (co-IP LC-MS) was performed to identify potential LASS2-interacting proteins in thyroid, breast, and liver cancer cell lines. Transcriptomics, proteomics and metabolomics analyses of multiple cancer cell types were performed using MS or LC–MS to further explore the underlying mechanisms involved. Among these tumour cells, the common LASS2 interaction partner transferrin receptor (TFRC) was analysed by protein–protein docking and validated by coimmunoprecipitation western blot, immunofluorescence, and proximity ligation assays. Then, we performed experiments in which tumour cells were treated with Fer-1 or erastin or left untreated, with or without inducing LASS2 overexpression, and assessed the molecular biological and cellular functions by corresponding analyses.

**Results:**

Low LASS2 expression is correlated with adverse clinical characteristic and poor prognosis in patients with thyroid cancer, breast cancer or HCC. Multiomics analyses revealed significant changes in the ferroptosis signalling pathway, iron ion transport and iron homeostasis. Our in vitro experiments revealed that LASS2 overexpression regulated ferroptosis status in these tumour cells by affecting iron homeostasis, which in turn inhibited tumour migration, invasion and EMT. In addition, LASS2 overexpression reversed the changes in tumour cell metastasis induced by either Fer-1 or erastin. Mechanistically, LASS2 interacts directly with TFRC to regulate iron homeostasis in these tumour cells.

**Conclusions:**

In summary, our study reveals for the first time that LASS2 can inhibit tumour cell metastasis by interacting with TFRC to regulate iron metabolism and influence ferroptosis status in thyroid, breast, and liver cancer cells, these results suggest potential universal therapeutic targets for the treatment of these cancers.

**Supplementary Information:**

The online version contains supplementary material available at 10.1186/s12935-024-03275-8.

## Introduction

Tumour metastasis is a major challenge in the clinical management of cancer, often limiting the clinical efficiency of drugs and resulting in poorer treatment outcomes. Metastasis limits clinical options and is a major cause of death in cancer patients [1–3]. The metastasis process is a complex molecular event involving multiple steps, genes and cells. Genes encoding on coproteins and tumour suppressors play critical yet opposing roles, and these molecules are often seen as potential therapeutic targets [4]. Accordingly, identifying the key molecules involved in metastasis can help us understand the mechanisms by which metastasis occurs, develop targeted drugs, and design new diagnostic or therapeutic approaches.

LAG1 longevity assurance homologue 2 (LASS2), also known as ceramide synthase 2 (CerS2), is localized to the endoplasmic reticulum and nuclear membrane structures [5]. LASS2 has been shown to act as a tumour suppressor in a variety of cancers, exhibiting different functions and molecular mechanisms in different tissue-specific cancers. For instance, LASS2 was found to cell proliferation and induce apoptosis in HepG2 hepatoblastoma cells (HBs) through downregulation of the NF-κB signalling pathway [5] and to suppress hepatocellular carcinoma (HCC) through upregulation of the TGF-β1-Smad4-PAI-1 axis [6]. Other studies have reported that LASS2 inhibits the migration and invasion of prostate [7], breast [8], and bladder cancer cells [9] which are associated with ATP60C and MMP2/MMP9 activity. LASS2-based mechanistic studies of heterogeneous and homogeneous states may lead to new strategies and more effective cancer treatment.

Ferroptosis, a newly identified type of oxidative cell death driven by the accumulation of reactive oxygen species (ROS), particularly iron-dependent phospholipid peroxidation, has been implicated in iron-and ROS-related diseases [10, 11]. Guan et al. [12] demonstrated that erastin-induced ferroptosis was caused by iron-mediated ROS production, which led to lipid peroxidation and cell death, inhibiting the invasion and migration of gastric cancer cells. Growing evidence indicates that ferroptosis is also strongly associated with tumorigenesis, progression, invasion, metastasis, drug resistance, and tumour immunity, and promoting ferroptosis is a potential therapeutic strategy [13–16]. Our previous study showed that LASS2 promotes mitochondrial ROS (mtROS) production by regulating mitochondrial function, possibly through the interaction between LASS2 and NDUFS2 (a core subunit of mitochondrial complex I) [17]. Recent studies have provided strong evidence that mitochondria play a central role in ROS generation and iron homeostasis and can induce or mediate the ferroptosis signalling pathway. However, whether LASS2 can affect tumour progression by regulating ferroptosis has not yet been reported.

Here, via bioinformatics analysis, we revealed heterogeneity in the expression of LASS2 in a variety of cancers. Based on the incidences of cancer, metastasis, and cancers previously reported to be associated with ferroptosis, we first verified that low levels of LASS2 were associated with poor clinical features and poor prognosis in patients with thyroid, breast, or liver cancer. In addition, for the first time, LASS2 overexpression was found to further inhibit cancer cell invasion and migration by regulating the ferroptosis signalling pathway in thyroid, breast, and hepatocellular cancer cells, possibly through direct interactions between LASS2 and TFRC.

## Materials and methods

### Bioinformatics analysis

Three public online databases, namely, the TMNplot (https://tnmplot.com/analysis/), TCGA (http://portal.gdc.cancer.gov), and GTEx (https://gtexportal.org/home/) databases, were used to analyse the transcriptome expression of LASS2 across cancers. The relationship between LASS2 expression and overall survival (OS) was analysed via the PanCanSurvPlot (https://smuonco.shinyapps.io/PanCanSurvPlot/), GEPIA (http://gepia.cancer-pku.cn), and Kaplan–Meier plotter (http://kmplot.com/analysis/index.php?p=background) tools. Gene Set Enrichment Analysis (GSEA) was performed via the TCGA database with the Clusterprofile package in R (v4.0.3) to investigate the functions of LASS2.

### Patient and tissue samples

Thyroid cancer, breast cancer, and HCC tissues were obtained from the Affiliated Hospital of Zunyi Medical University. In this study, 35 thyroid cancer, 30 breast cancer, and 38 HCC tissue samples were collected and embedded in paraffin. Informed consent was obtained from all patients who provided tissue samples, and the study was approved by the Ethics Committee of Zunyi Medical University [(2020) 1-150, KLL-2019-020]. This study was conducted in accordance with the Declaration of Helsinki.

### Immunohistochemistry (IHC)

IHC was performed as described previously [18]. The following primary antibodies were used: anti-LASS2 (sc-390745, 1:200; Santa Cruz Biotechnology, Santa Cruz, CA, USA) and anti-TFRC (ab214039, 1:200; Abcam, Cambridge, UK). The staining scores for LASS2 and TFRC were determined using a specific intensity distribution (ID) score as follows: percentage of positive cells (0: < 5%; 1: 5–25%; 2: 26–50%; 3: 51–75%; 4: 76–100%), as determined by the product of the staining intensity grading (0, negative; 1, weak; 2, moderate; 3, strong). The ID score was calculated by multiplying these two factors: in general, a score of ≤ 4 was considered to indicate low expression, and a score of ≥ 6 was considered to indicate high expression.

### Transcriptomics

Total RNA from BCPAP cells transfected with Adv-GFP or Adv-*h*LASS2-GFP was extracted with a Buffer RLT Plus Kit (QIAGEN) and purified using a RNeasy spin column. Sequencing was performed by BGI Company (Shenzhen, China) on an RNAref + RNAseq Illumina platform. The raw sequencing data were filtered using SOAPnuke (v1.5.6), differential gene expression was detected using DESeq2R (v1.4.5), and differentially expressed genes were subjected to Gene Ontology (GO) analysis and Kyoto Encyclopedia of Genes and Genomes (KEGG) enrichment analysis.

### Proteomics data analyses

The protein samples were submitted to BGI Company (Shenzhen, China) for proteomics analysis. Briefly, protein samples from BCPAP cells transfected with Adv-GFP or Adv-*h*LASS2-GFP were extracted with lysis buffer and quantified via the Bradford assay. For digestion, trypsin was added to the protein sample at a mass ratio of 1:20 (trypsin to protein). The tryptic peptides were detected by high-resolution liquid chromatography-mass spectrometry (LC–MS/MS). The LC‒MS/MS data were processed using the MaxQuant integrated Andromeda search engine (v. 2.1.3.0). The UniProtKB/Swiss-Prot subset was used to identify proteins in the corresponding species. Functional analysis was performed on the differentially enriched proteins based on the quantitative results.

### Cell culture and transfection

The human papillary thyroid carcinoma cell line BCPAP, the mouse HCC cell line Hepa1-6, and the human HB cell line HepG2 were obtained from the Stem Cell Bank of the Chinese Academy of Sciences (Shanghai, China). BCPAP cells were cultured in 1640 medium supplemented with 10% foetal bovine serum (FBS), 1% NEAA, 1% GlutaMAX, and 1% 100 mM sodium pyruvate solution. The Hepa1-6 and HepG2 cells were cultured in Dulbecco’s modified Eagle medium (DMEM) supplemented with 10% FBS. All the cell lines were cultured at 37 °C, 5% CO_2_, and 100% humidity in a special incubator. The human triple-negative breast carcinoma cell line MDA-MB-231 was obtained from Feng Hui Biotechnology (Changsha, Hunan, China) and cultured in L-15 medium supplemented with 10% FBS at 37 °C and 100% humidity under defined culture conditions. All cells were identified using short tandem repeats, and mycoplasma contamination was excluded.

Cells were infected with Adv-GFP, recombinant adenovirus Adv-*h*LASS2-GFP, or Adv-*m*LASS2-GFP for 48 h. The cancer cells were then divided into negative control (NC), null (Adv-GFP), and experimental (Adv-LASS2-GFP) groups. Cells treated with ferrostatin-1 (Fer-1; HY-100579, 1 μM) or erastin (HY-15763, 5 μM) or left untreated for 24 h before infection were divided into the following four groups: Adv-GFP, Adv-LASS2-GFP, Adv-GFP + Fer-1/erastin, and Adv-LASS2-GFP + Fer-1/erastin.

### Analysis by reverse transcription-quantitative polymerase chain reaction (RT-qPCR) and western blot

RT-qPCR and western blotting procedures were performed as described previously [17]. Briefly, the sequences of the LASS2 and GAPDH primers are shown in Additional file 1: Table S1. The following primary antibodies were used: LASS2 (sc-390745, 1:1,000; Santa Cruz), TFRC (#13113, 1:1,000; Cell Signaling Technology), ferritin heavy chain 1 (FTH1; #29650, 1:1,000; Cell Signaling Technology), ferritin light chain (FTL; #10727-1-AP, 1:1,000; Proteintech), GPX4 (#52455, 1:1,000; Cell Signaling Technology), E-Cadherin (ET1607-75, 1:5,000; HuaBio), N-Cadherin (ET1067-37, 1:1,000; HuaBio), vimentin (#10366-1-AP, 1:2,000; Proteintech), Snail (#1309-1-AP, 1:1,000; Proteintech), and Slug (sc-166476, 1:800; Santa Cruz). The following secondary antibodies were used: goat anti-rabbit IgG (HA1001, 1:5000; HuaBio) and goat anti-mouse IgG (HA1006, 1:5000; HuaBio).

### Transmission electron microscopy

Cells in the logarithmic growth phase were inoculated into T25 vials and infected with either Adv-GFP, Adv-*h*LASS2-GFP or Adv-*m*LASS2-GFP for 48 h. The cells were then gently washed 1–2 times with phosphate-buffered saline (PBS), detached using a cell scraper in a consistent direction, and collected via centrifugation. Subsequently, the cells were fixed with 2.5% glutaraldehyde fixative overnight at 4 °C. The samples were then pre-embedded in low melting point agarose. After three washes with 0.1 M phosphate buffer (pH 7.2), the samples were fixed with 1% osmotic acid for 2 h at 4 °C, and then re-embedded in low-melting-point agarose. Next, the samples were dehydrated using an ethanol gradient. Subsequently, the samples were permeabilized and placed in a 60 °C oven for 48 h for polymerization. Ultrathin sections were obtained and counterstainied with 3% uranyl acetate and 2.7% lead citrate. Finally, alterations in the mitochondrial morphology of the cells were observed via transmission electron microscopy (HT7800; Hitachi, Tokyo, Japan).

### Malondialdehyde (MDA) measurement

Cells in the logarithmic growth phase were inoculated into T25 vials and infected with either Adv-GFP or Adv-*h*LASS2-GFP or Adv-*m*LASS2-GFP for 48 h. The cells were collected for sample preparation via western blotting and immunoprecipitation (IP), and cell lysis buffer (P0013; Beyotime, Shanghai, China) was added to lyse the cells via ultrasonication (power, 200 W; ultrasound, 3 s; interval, 10 s; repeated 30 times) on ice. The supernatant was removed by centrifugation at 2000×*g* for 10 min, and an appropriate amount of supernatant was used for bicinchoninic acid protein quantification. Then, 100 μL of the supernatant was mixed with 200 μL of thiobarbituric acid working solution, and the mixture was heated in a water bath for 30 min. Afterwards, the above mixture was cooled using running water and centrifuged at 1000×*g* for 5 min, after which the absorbance was measured at 532 nm.

### Mitochondrial reactive oxygen species (ROS) detection

Cells in the logarithmic growth phase were inoculated into T25 vials and infected with either Adv-GFP or Adv-*h*LASS2-GFP for 48 h. EDTA-free trypsin was added for cell digestion. MitoSOX™ Red (Thermo Fisher Scientific, Waltham, MA, USA) solution was prepared under low light conditions. Then, 500 μL of working solution was added to each sample, which was subsequently placed in an incubator at 37 °C for 10 min. Final detection was performed via a flow cytometer (BD Biosciences, Franklin Lakes, NJ, USA) at excitation wavelengths of 490–510 nm (green) and emission wavelengths of 570–600 nm (red).

### Transwell migration and invasion assays

Cell migration and invasion capacity were measured via transwell assays using a polycarbonate membrane in a transwell chamber (Corning, NY, USA). Migration was assessed by using an unincorporated matrix gel. The transwell chambers were placed in a 24-well plate. In the lower chamber, 500 μL of complete medium containing 20% FBS was added, and the upper chamber was filled with 5 × 10^4^ cells/100 μL of serum-free medium. The plate was then incubated at 37 °C for 48–72 h. Next, the transwell chambers were stained with crystal violet for 3 min. The cells in the upper chamber of the transwell were washed with PBS and allowed to air dry. Finally, images were taken via microscopy using three different fields of view for each chamber. Matrix gel was added to measure the cell invasion ability, and the rest of the steps were with the same as in the migration measurement.

### Cell counting kit-8 (CCK-8) assay

BCPAP, MDA-MB-231 and Hepa1-6 cells were inoculated into 96-well plates. A total of 2 × 10^3^ cells were added to each well in 100 µL of complete medium and then incubated continuously at 37 °C with 5% CO_2_ for 48 h. Ten microlitres of CCK-8 solution (HY-K0301, MedChemExpress) was added to each well, and the cells were incubated for 1.5 h in the dark. Cell viability was measured by absorbance at 450 nm.

### ***Fe***^***2***+^***measurement***

The cells were inoculated into 24-well plates or T25 bottles and infected with either Adv-GFP or Adv-LASS2-GFP for 48 h. After washing three times with PBS, 1 μM FerroOrange (F374; Tong Ren Chemistry, West Lafayette, IN, USA) working solution was added, and the cells were placed in a cell incubator at 37 °C for 30 min. The signals were then measured at excitation wavelengths of 561 nm and emission wavelengths of 570–620 nm using an ortho-fluorescence microscope and a flow cytometer, respectively.

### Molecular docking

ZDOCK v3.0.2 was used to predict the binding patterns of the individual combinatorial proteins. Before docking started, protein structure files were obtained from the UniProt database. These proteins were subsequently processed using PyMOL v2.5.2, to eliminate water molecules and hydrogen atoms, as well as nontarget structural proteins. Docking analysis was performed with the default settings of ZDOCK v3.0.2 for global rigid docking. The protein–protein binding affinity was obtained based on docking scores.

### Immunofluorescence

Cells in the logarithmic growth phase were seeded onto 24-well culture plates. After fixation with 4% paraformaldehyde for 30 min, the cells were permeabilized with 1% Triton X-100 for 20 min. Subsequently, the cells were blocked with 10% serum for 1 h, followed by overnight incubation at 4 °C with primary antibody applied dropwise. The next day, goat anti-mouse IgG H&L (Alexa Fluor® 647, ab150115, 1:100; Abcam, Cambridge, UK) and fluorescein (FITC)-AffiniPure donkey anti-rabbit IgG (H+L) (#711–095-152, 1:100; Jackson ImmunoResearch, West Grove, PA, USA) were added and incubated for 2 h at 25 °C. The nuclei were stained with Hoechst 3342 (Cell Signaling Technology), after which a fluorescent quencher was added dropwise. The final observation was performed under a fluorescence microscope with LASS2 in red, TFRC in green, and DAPI in blue.

### Co-IP and liquid chromatography-mass spectrometry (LC–MS)/western blot analysis

BCPAP, MDA-MB-231, and Hepa1-6 cells were transfected with Adv-GFP, Adv-*h*LASS2-GFP, or Adv-*m*LASS2-GFP, and subsequently lysed using ice-cold IP lysis/wash buffer (Thermo Fisher Scientific). The lysates were collected in centrifuge tubes and centrifuged at 13,000×*g* for 10 min. The protein concentration was determined using a bicinchoninic acid protein quantification kit. GFP Nanoselector beads (NB Biolabs, Chengdu, China) were added to the supernatant, which was subsequently incubated for 60 min at 4 °C with rotation. The encapsulated GFP Nanoselector beads were resuspended in SDS-PAGE buffer, followed by boiling at 95 °C for 10 min. Afterwards, the mixture was centrifuged at 2500×*g* for 2 min at 4 °C. The resulting supernatant was subsequently subjected to SDS‒PAGE or western blot analysis.

To identify proteins that interact with LASS2, co-IP samples were subjected to SDS-PAGE and stained with Coomassie blue (Epizyme, Cambridge, MA, USA). Specific bands were excised and analysed via LC–MS. The Go STRING and KEGG pathway databases were used to identify the potential interaction networks.

Additionally, co-IP samples from the BCPAP, MDA-MB-231, Hepa1-6, and HepG2 cells transfected with Adv-GFP, Adv-*h*LASS2-GFP or Adv-*m*LASS2-GFP were analysed via western blot analysis to verify the interaction between LASS2 and TFRC.

### Proximity ligation assay (PLA)

The interaction between LASS2 and TFRC was analysed using a proximity linkage assay (DUO92101; Sigma-Aldrich, St. Louis, MO, USA) according to the manufacturer’s instructions. Briefly, appropriate cells were seeded on culture plates and incubated overnight at 37 °C. The first day of processing was consistent with the immunofluorescence protocol. The next day, the cells were washed three times with Buffer A for 5 min each and incubated with secondary antibody for 30 min at 37 °C. After washing, ligase was added, and ligation was performed at 37 °C for 1 h. Then, polymerase was added, and amplification was performed at 37 °C for 100 min. Finally, the cells were washed and blocked with a blocking solution containing DAPI. The results were analysed using an ortho-fluorescence microscope (Leica SP, Wetzlar, Germany) or Airyscan laser confocal microscopy (LSM 900, CARL ZEISS, Germany).

### Overall metabolomics

The metabolites were extracted from MDA-MB-231 cells transfected with Adv-GFP or Adv-*h*LASS2-GFP via the assistance of MetWare (Wuhan, China). In brief, hydrophilic and hydrophobic compounds were separately extracted and analysed using an LC–ESI–MS/MS system (UPLC, ExionLC AD, https://sciex.com.cn/; MS, QTRAP® System, https://sciex.com/) in accordance with previously published methods [19]. The mass spectrometry data were processed by Software Analyst 1.6.3. The highly enriched metabolic pathways associated with the differentially abundant metabolites were identified using KEGG and metabolite set enrichment analysis (MSEA).

### Statistical analysis

All the statistical analyses were performed using SPSS Statistics v29.0. Western blot greyscale values were analysed using Gelpro v32. Figures were created using GraphPad Prism v8. A t test was used for comparisons between two groups, and analysis of variance was used for comparisons between multiple groups. The results are expressed as the mean ± standard deviation (mean ± SD), and *P* < 0.05 was considered to indicate a statistically significant difference. All the experiments were independently repeated three times.

## Results

### LASS2 is a novel prognostic marker in human carcinomas

**Fig. 1:**
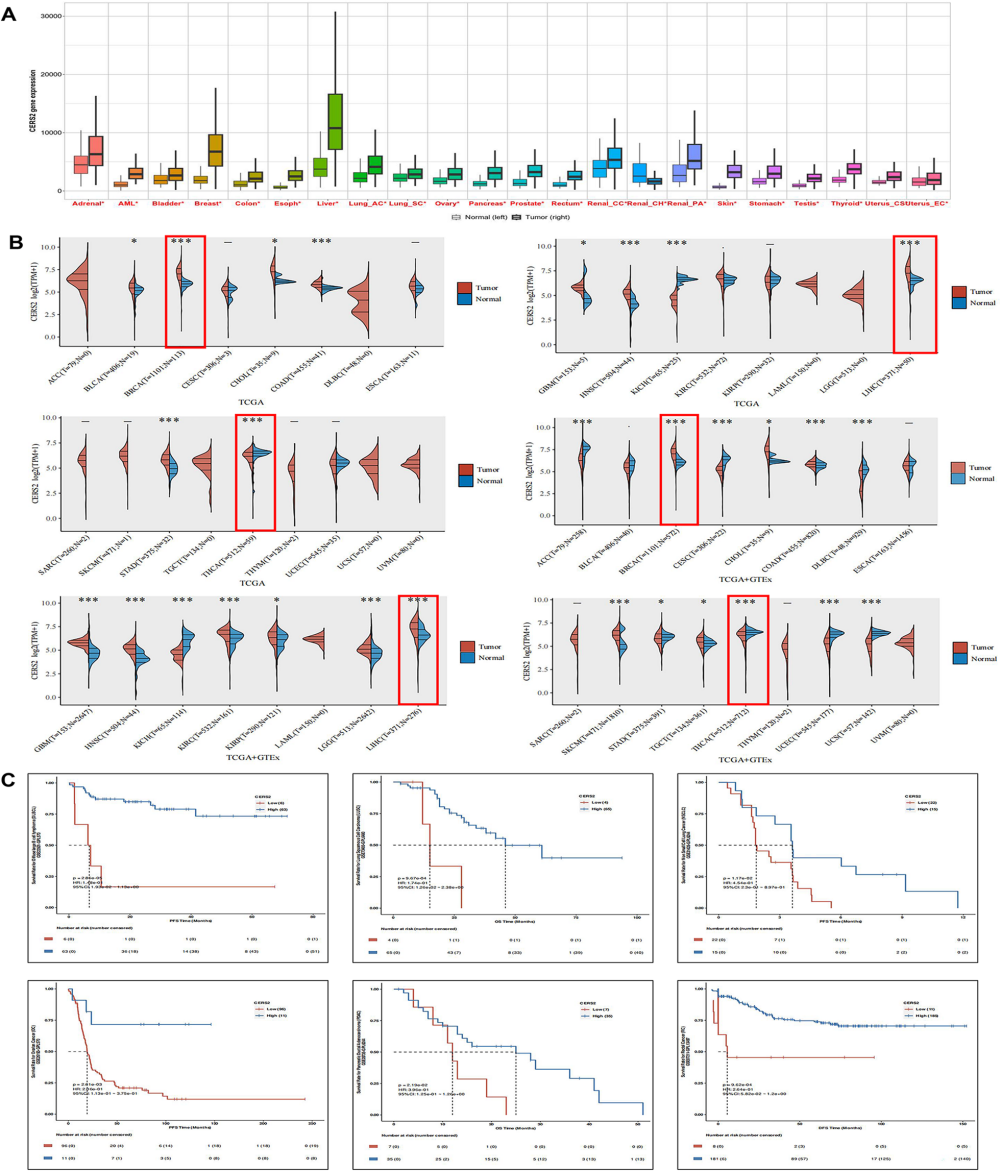
Multitumour analysis of the prognostic value of LASS2 expression in human tumours. **A** TMNplot analysis of LASS2 expression in normal and tumour tissues. The left side represents normal tissues, and the right side represents tumour tissues. **B** TCGA database and TCGA + GTEx combination database assessment of LASS2 expression in multiple tumours. Red indicates tumour tissue (T), and blue indicates normal tissue (N). **C** The prognostic value of LASS2 across cancers was analysed via the PanCanSurvPlot database. High expression of LASS2 predicted high overall survival in patients with DLBCL, LUSC, NSCLC, OC, PDAC or rectal cancer (RC). *P < 0.05, **P < 0.01, ***P < 0.001

Using several online databases, we evaluated the levels of LASS2 in patients with various cancers and its potential impact on patient survival. The TMNplot database contains information on 22 types of carcinomas, and LASS2 expression was upregulated in 21 tumour types but downregulated in 1 tumour type (Fig. 1A). The TCGA database analysis revealed information for 25 types of carcinomas, with LASS2 expression upregulated in 23 types of carcinomas but downregulated in 2 types of carcinomas. The combined TCGA and GTEx analyses showed that LASS2 expression was upregulated in 16 carcinomas and downregulated in 9 carcinomas (Fig. 1B). In addition, the pan-cancer prognostic analysis of the PCSP database revealed that among the tumours in which high expression of LASS2 predicted good OS in patients, the most significantly different tumours were diffuse large B-cell lymphoma (*P* = 2.86e−05), lung squamous cell carcinoma (*P* = 9.67e−04), non-small cell lung cancer (*P* = 1.17e−02), ovarian cancer (*P* = 2.61e−03), pancreatic ductal adenocarcinoma (*P* = 2.19e−02), or rectal cancer (*P* = 9.62e−04) (Fig. 1C). These results indicate that LASS2 is differentially expressed between normal and tumour tissues in various cancers and is correlated with patient prognosis, suggesting that LASS2 may be a novel diagnostic marker in human carcinomas.

### A low level of LASS2 is associated with adverse clinical characteristics in thyroid cancer, breast cancer and HCC and indicates a poor prognosis

**Fig. 2:**
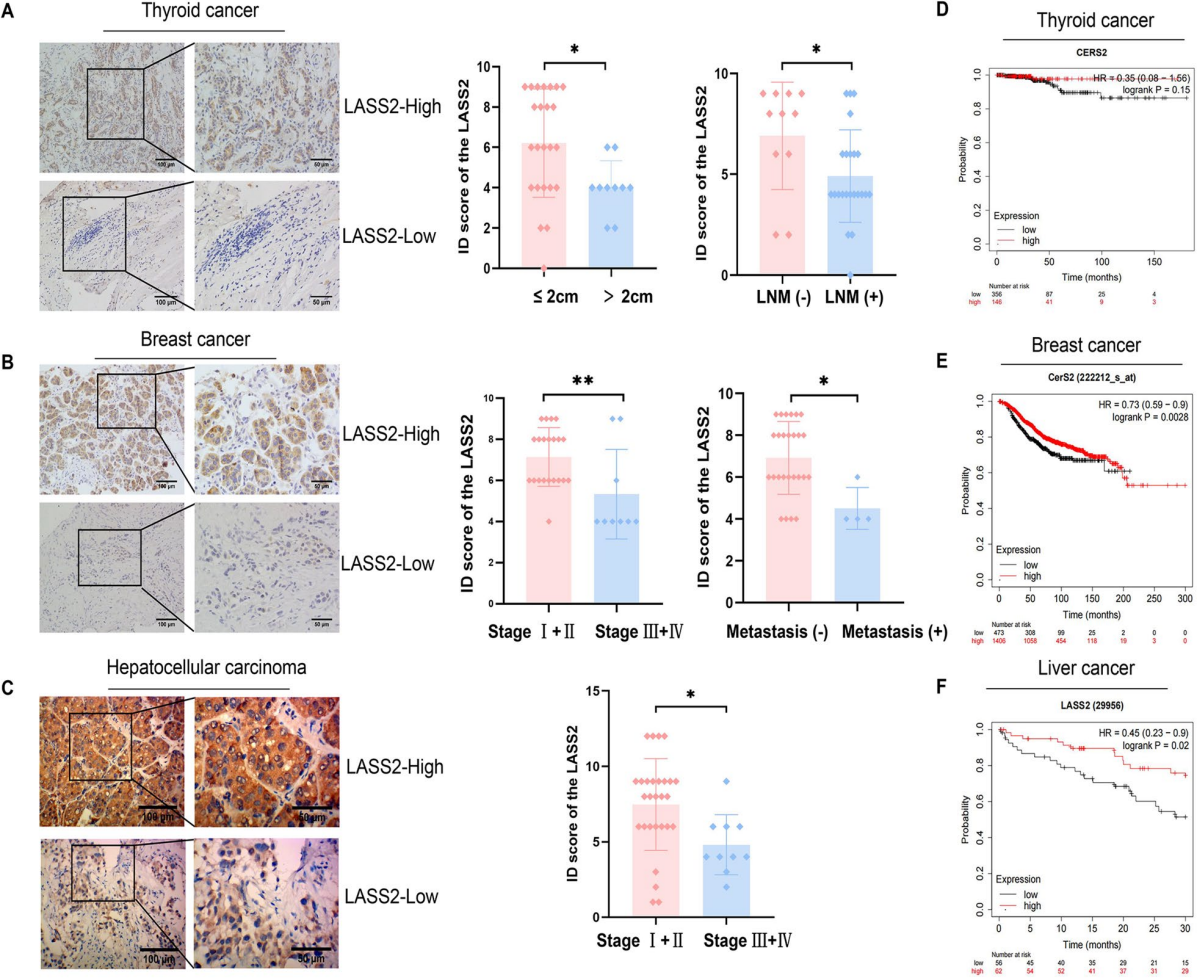
LASS2 expression is correlated with clinical parameters and the prognosis of thyroid, breast, and liver cancer. **A**–**C** LASS2 expression in thyroid, breast, and liver cancer tissues is correlated with tumour size, lymph node metastasis, and clinical TNM stage (scale bar: 100/50 μm). **D**–**F** Public database analysis of survival differences between patients with high and low LASS2 expression in thyroid, breast, and liver cancers. *P < 0.05, **P < 0.01

Based on the results of the TCGA and GTEx data analyses (Fig. 1B) and previous studies reporting that the mRNA levels of LASS2 during cell signalling processes do not always correspond to protein levels or enzyme activity [20, 21], we selected the carcinomas with the most significant differences in LASS2 expression (Fig. 1B) to explore the correlation between the LASS2 protein level and prognosis. Therefore, we used IHC to examined the LASS2 protein levels in thyroid cancer, breast cancer and HCC tissues. The clinicopathological information of the patients is shown in Table 1. The results showed that in thyroid cancer, the LASS2 protein level was negatively correlated with lymph node metastasis (*P* = 0.03) and tumour size (*P* = 0.023) (Fig. 2A). The LASS2 protein level was negatively correlated with clinical TNM stage in both breast cancer and HCC tissues (*P* = 0.003 and *P* = 0.014, respectively) (Fig. 2B and C), and negatively correlated with distant metastasis in breast cancer (*P* = 0.031) (Fig. 2B). In addition, we analysed the relationship between LASS2 mRNA levels and the prognosis of patients with these three tumour types using public databases. The results showed that high-risk levels of LASS2 correlated with better survival outcomes (Fig. 2D–F). These results further confirmed that LASS2 plays a tumour suppressor role in thyroid cancer, breast cancer and HCC.

**Table 1:** Correlation between LASS2 expression and clinicopathological parameters in three tumors

Characteristic	LASS2 ID score										
	Thyroid cancer				Breast cancer				Hepatocellular carcinoma		
	0–4	6–12	*P* value		0–4	6–12	*P* value		0–4	6–12	*P* value
Sex			0.366	Age (years)			0.597	Sex			0.63
Male	7	10		< 51	4	14		Male	6	25	
Female	9	8		≥ 51	3	9		Female	4	3	
Age (years)			0.652	Tumor size			0.236	Age (years)			0.91
< 55	14	16		≤ 2 cm	0	5		< 50	1	11	
≥ 55	2	2		> 2 cm	7	18		≥ 50	9	17	
Tumor size			0.023*	TNM stage			0.003*	TNM stage			0.014*
≤ 2 cm	8	16		I	0	2		I	0	5	
> 2 cm	8	2		II	1	18		II	4	19	
LNM			0.030*	III	3	2		III	6	3	
Negative	2	9		IV	3	1		IV	0	1	
Positive	14	9		Metastases			0.031*				
				Negative	3	1					
				Positive	4	22					
				LNM			0.143				
				Negative	3	17					
				Positive	4	6

### LASS2 is closely associated with the ferroptosis-related pathway in multiple cancer cell lines

**Fig. 3:**
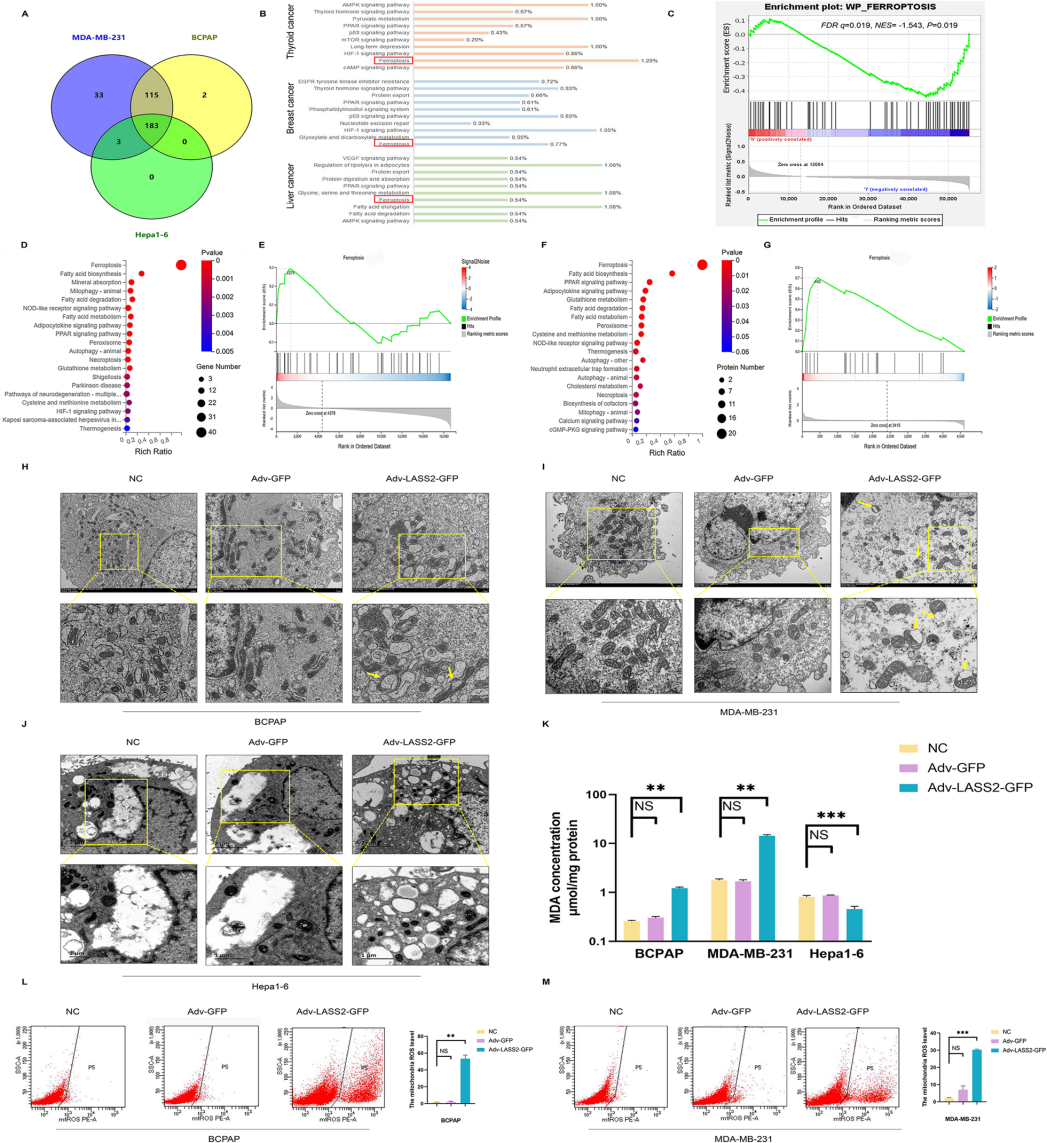
Effects of LASS2 overexpression on ferroptosis in different tumours. **A** Venn diagram of 183 overlapping signalling pathways enriched in LASS2-overexpressing cells according to LC–MS analysis. **B** Important signalling pathways based on Venn diagrams. **C** The ferroptosis signalling pathway was enriched based on Gene set enrichment analysis (GSEA) from LASS2 overexpression (*FDR q* = 0.019, *NES* = -1.543, *P* = 0.019). **D**–**G** Transcriptomics (**D**) and proteomics (**F**) analyses of the enriched KEGG pathways. GSEA in (**E**) and (**G**) reveals enriched pathways for differentially expressed genes. **H**–**J** Morphological changes in mitochondria in three tumour cell lines were observed via transmission electron microscopy. The typical features are indicated by the yellow arrows. **K** MDA content was measured by a colorimetric assay after LASS2 overexpression in the BCPAP, MDA-MB-231, and Hepa1-6 cell lines. **L**, **M** Mitochondrial ROS levels measured by flow cytometry. **P < 0.01, ***P < 0.001

Dysregulation of LASS2 gene transcription is associated with tumour development, and its activity is regulated by multiple protein–protein interactions [21]. To screen potential LASS2-interacting factors and regulatory signalling pathways, we performed co-IP-coupled LC–MS analysis of the human papillary thyroid carcinoma cell line BCPAP, the human triple-negative breast carcinoma cell line MDA-MB-231, and the mouse HCC cell line Hepa1-6 cells. The overexpression efficiency of LASS2 in these cells was confirmed by RT qPCR and western blot (Additional file 2: Fig. S1A–C). Co-IP-LC/MS analysis revealed differences and similarities in LASS2-interacting proteins among these tumour cell lines, indicating distinctions in genetic backgrounds and tumour types. As shown in Fig. 3A, Venn diagrams illustrate the shared and distinct signalling pathway changes in the three tumour cell lines compared to those in the control. Interestingly, further pathway analysis revealed that LASS2-interacting proteins were involved in similar signalling pathways, such as the ferroptosis, p53, mTOR, and HIF-1 pathways (Fig. 3B). HIF-1 and p53 signalling pathways were reported that regulated iron uptake [22] or cystathionine levels [23], impacting susceptibility to ferroptosis.

As mentioned earlier, ferroptosis is a newly identified form of programmed cell death dependent on ROS and iron [24, 25]. Previous investigations have shown the functionality of LASS2 in mitochondrial and ROS production [5, 17, 26]; interestingly, our findings now show that LASS2-interacting proteins are also involved in the ferroptosis signalling pathway. To elucidate the downstream signalling pathways resulting from LASS2 overexpression, we performed GSEA using the TCGA dataset and found that the ferroptosis signalling pathway was enriched (Fig. 3C) (*FDR q* = 0.019, *NES* = − 1.543, *P* = 0.019). In addition, we explored the KEGG pathway and. GSEA results from the transcriptomics and proteomics data of BCPAP cells for further verification. The ferroptosis pathway was significantly enriched in LASS2-overexpressing BCPAP cells (Fig. 3D, F). Moreover, the GSEA results indicated that the ferroptosis signalling pathway was obviously enriched in LASS2- overexpressing BCPAP cell group compared with the control group (Fig. 3E, G). The above data suggest that there is a link between LASS2 expression and the ferroptosis pathway, which needs to be further explored.

### Overexpression of LASS2 regulates the status of ferroptosis in multiple tumour cell lines

To assess the effect of LASS2 on ferroptosis, we used transmission electron microscopy (TEM) to observe the morphological changes in mitochondria in thyroid cancer, breast cancer, and HCC cell lines. In BCPAP (Fig. 3H) and MDA-MB-231 cells (Fig. 3I), we observed outer mitochondrial membrane rupture, as well as crumpled mitochondria or mitochondrial crista loss—and other morphologic alterations not shown in this report, suggesting the involvement of multiple regulatory mechanisms. However, we observed no typical morphological characteristics of ferroptosis in Hepa1-6 cells overexpressing LASS2 (Fig. 3J). As the accumulation of MDA (an end-product of polyunsaturated fatty acid peroxidation) and elevated ROS (oxidative stress factor) are hallmarks of ferroptosis [27], we next investigated the effect of LASS2 overexpression on MDA and ROS levels. As shown in Fig. 3K, the MDA levels were significantly increased by LASS2 overexpression in BCPAP and MDA-MB-231 cells but were decreased in Hepa1-6 cells. In our previous study, we reported that LASS2 overexpression significantly increased ROS levels in HepG2 HB and Hepa1-6 cells. Here, using flow cytometry, we found that mitochondrial ROS production was significantly increased in BCPAP and MDA-MB-231 cells overexpressing LASS2 (Fig. 3L, M). The above results demonstrated that LASS2 overexpression promoted ferroptosis in BCPAP and MDA-MB-231 cells but inhibited ferroptosis in Hepa1-6 cells. These findings suggest that LASS2 has differential effects on the regulation of ferroptosis between tumour types.

### LASS2 directly interacts with TFRC in multiple tumour cells

**Fig. 4:**
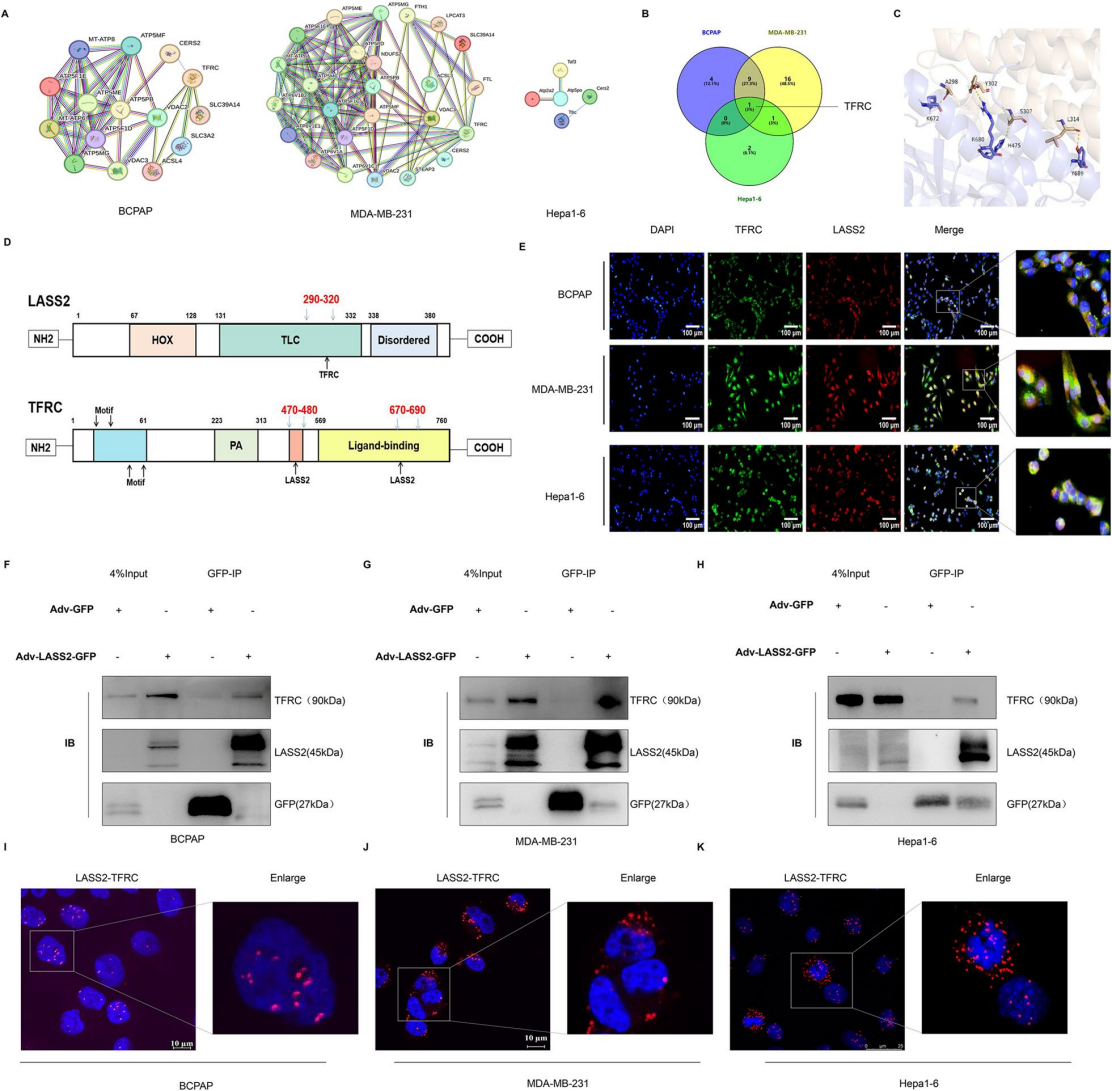
The interaction between LASS2 and TFRC. **A** Network interaction maps constructed using STRING software (https://cn.string-db.org/) for enriched ferroptosis-related proteins in the tumour cell lines revealed by LC–MS. (Only some of the proteins are presented.) **B** The Venn diagram with TFRC in the intersection of the three tumours.** C** Prediction of LASS-TFRC protein binding by ZDOCK 3.0.2. **D** The binding mode of the LASS2-TFRC complex was predicted via ZDOCK 3.0.2, and its structural domains were identified via UniProt (https://www.uniprot.org/). **E** Detection of the colocalization of endogenous LASS2 with TFRC by immunofluorescence in the BCPAP, MDA-MB-231, and Hepa1-6 cell lines (scale bar: 100/50 μm). **F**–**H** Interaction of LASS2 with TFRC, revealed by co-IP and western blotting in three cell lines. **I**–**K** The direct interaction between LASS2 and TFRC was confirmed by PLA

Our previous study showed that LASS2 can regulate ferroptosis across multiple tumour cell lines; however, the underlying mechanism for this phenotype is still unclear. Using a protein co-IP-coupled LC–MS assay and analysing interaction networks associated with the ferroptosis signalling pathway via STRING in LASS2-overexpressing tumour cell lines (BCPAP, MDA-MB-231, and Hepa1-6 cells), we identified candidate LASS2-interacting proteins. As shown in Fig. 4A and B, TFRC was unexpectedly identified as a candidate. To further verify whether there is mutual binding between LASS2 and TFRC, we first performed molecular docking analysis of these two proteins. The results showed a binding affinity of − 16.1 kcal/mol for LASS2 and TFRC, indicating their possible interaction (Fig. 4C).

By using ZDOCK 3.0.2 to predict functional protein domains, a probable interaction was identified between LASS2 and TFRC through the TLC domain (amino acids 290–320) (Fig. 4D). Next, immunofluorescence experiments were used to confirm the colocalization of endogenous proteins, and the results showed that endogenous LASS2 colocalized with TFRC in all three cell types (Fig. 4E). Importantly, this interaction was confirmed by a co-IP-western blot (Fig. 4F–H), and the results were consistent with the molecular docking prediction. These findings were further confirmed using PLA (Fig. 4I–K). Overall, we found for the first time that LASS2 directly interacts with TFRC in thyroid, breast, and liver cancer cell lines.

### LASS2 overexpression affects ferroptosis by regulating iron homeostasis

**Fig. 5:**
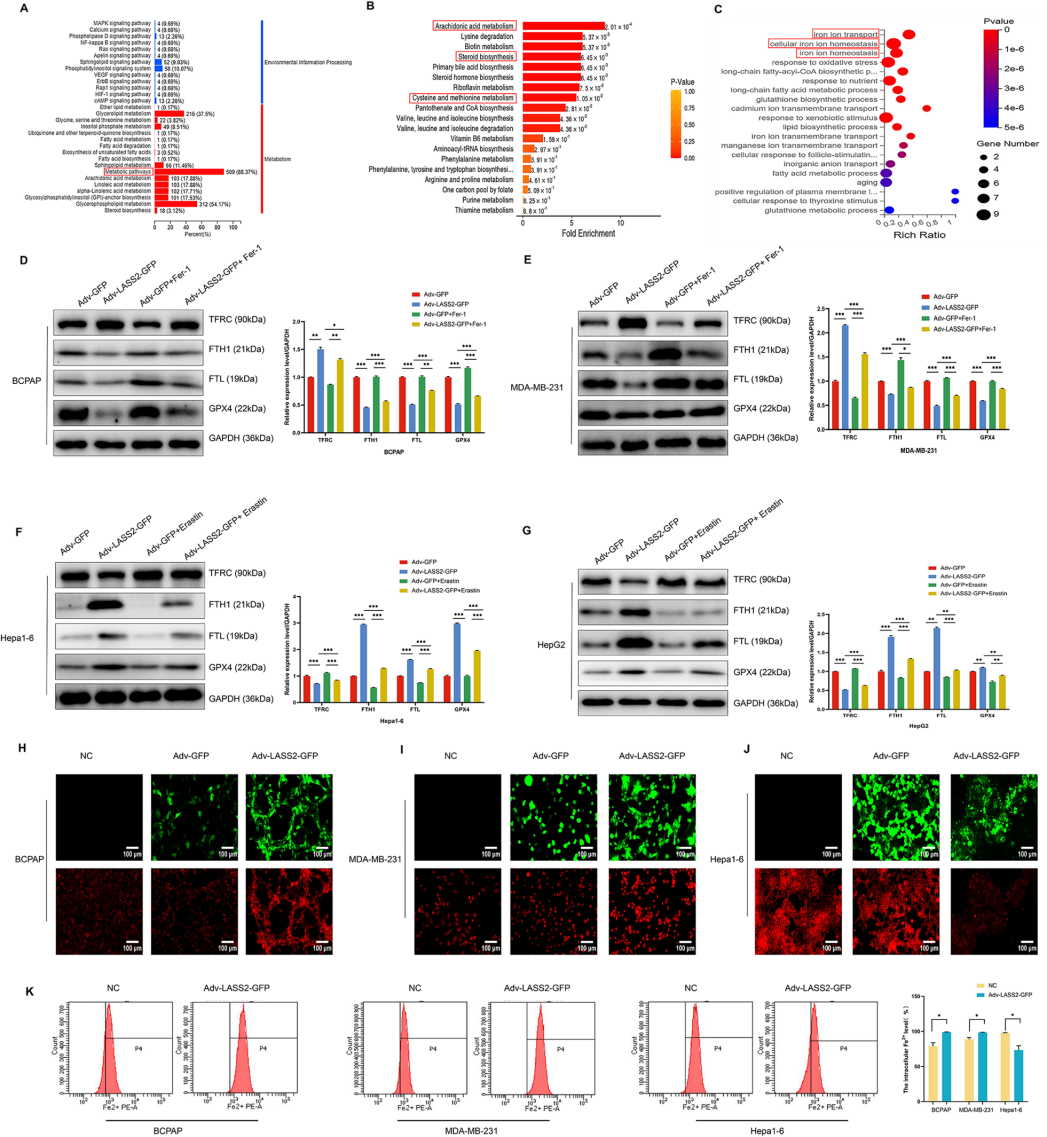
LASS2 overexpression alters the ferroptosis status of different cell lines through dysregulation of iron homeostasis. **A** KEGG categorization plot of differentially abundant metabolites. The y-axis shows the name of the KEGG metabolic pathway, and the x-axis shows the number of differentially abundant metabolites under the altered pathway, with their number as a proportion of the total number of metabolites annotated. **B** Metabolite set enrichment analysis (MSEA) of the metabolic datasets. **C** KEGG enrichment scatterplot of differentially expressed genes. **D**–**G** Changes in the expression of key proteins involved in ferroptosis and iron homeostasis in different tumour cell lines, as determined by western blotting. **H**–**K** The intracellular Fe^2+^ concentration was measured via fluorescence imaging and flow cytometry (scale bar: 100/50 μm). *P < 0.05, **P < 0.01, ***P < 0.001

To further explore the underlying mechanism, we performed metabolomic analysis of MDA-MB-231 cells, and the KEGG results revealed significant enrichment of pathways, including metabolic pathways (Fig. 5A). In addition, MSEA enrichment analysis revealed enrichment in pathways such as arachidonic acid metabolism, steroid biosynthesis and cysteine and methionine metabolism, all of which are closely associated with ferroptosis (Fig. 5B). Most notably, further transcriptomics analysis revealed significant enrichment of signalling pathways related to iron ion transport and iron homeostasis (Fig. 5C). TFRC, as a major regulator of iron uptake in cells, is involved in the regulation of iron metabolism and serves as a specific marker of ferroptosis [28]. Therefore, we first evaluated the effect of LASS2 on the TFRC protein level in multiple tumour cell lines with or without treatment with a ferroptosis inhibitor (Fer-1) treatment or a ferroptosis agonist (erastin). The results indicated that LASS2 overexpression significantly upregulated TFRC expression in BCPAP and MDA-MB-231 cells (Fig. 5D and E) but downregulated TFRC expression in Hepa1-6 and HepG2 cells (Fig. 5F, G). Interestingly, LASS2 overexpression reversed the changes in TFRC levels induced by either Fer-1 or erastin in these tumour cell lines (Fig. 5D–G).

FTH1 and FTL are important iron storage proteins and are key players in the ferroptosis signalling pathway, which is triggered by an imbalance in iron homeostasis and ROS levels. The concentration of free iron is altered by the inhibition of GPX4 [29], which is an antioxidant enzyme and a significant negative regulator of ferroptosis [30, 31]. Next, we further investigated the effect of LASS2 on the levels of FTH1, FTL, and GPX4. Western blot analysis confirmed that LASS2 overexpression decreased the levels of FTH1, FTL, and GPX4 in BCPAP and MDA-MB-231 cells (Fig. 5D, E), but different results were obtained in Hepa1-6 and HepG2 cells (Fig. 5F, G). Likewise, LASS2 overexpression reversed the changes in the levels of FTH1, FTL and GPX4 induced by either Fer-1 or erastin in these tumour cell lines (Fig. 5D–G). Furthermore, we examined the effect of LASS2 on the intracellular Fe^2+^ concentration via fluorescence imaging and flow cytometry, and the results indicated that LASS2 overexpression increased the intracellular Fe^2+^ concentration in BCPAP and MDA-MB-231 cells and decreased the intracellular Fe^2+^ concentration in Hepa1-6 cells (Fig. 5H–K). Collectively, these results confirmed that LASS2 can affect ferroptosis by regulating iron homeostasis in multiple tumour cell lines.

### Overexpression of LASS2 inhibits the metastasis of multiple tumour cell lines through ferroptosis

**Fig. 6:**
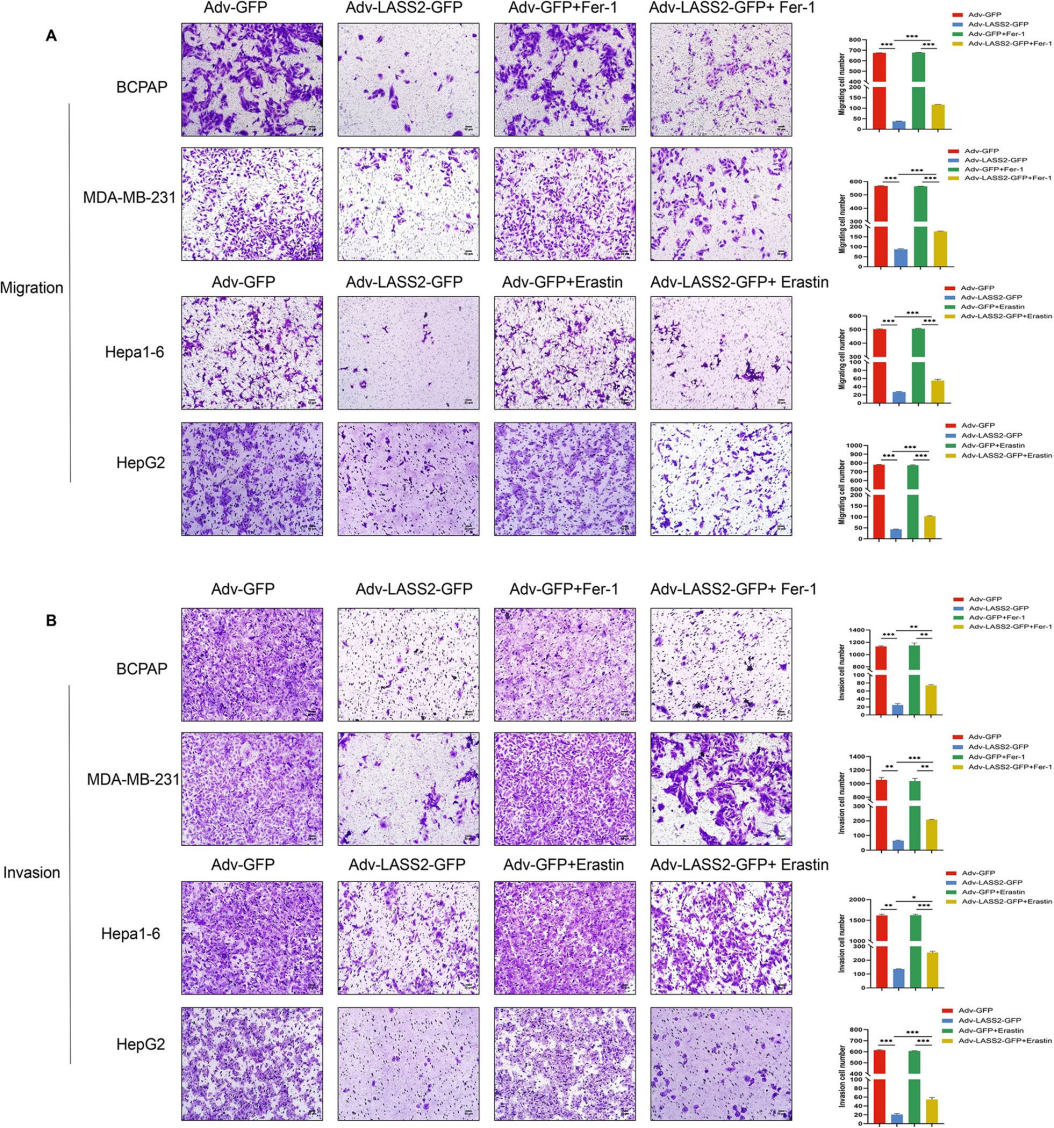
LASS2 overexpression inhibits the invasion and migration of tumour cell lines by mediating ferroptosis. (**A**) Migration and (**B**) invasion ability of BCPAP, MDA-MB-231, Hepa1-6, and HepG2 cells determined by transwell assay. *P < 0.05, **P < 0.01, ***P < 0.001

Ferroptosis plays a pivotal role in regulating the initiation, invasion, and metastasis of multiple cancers [32, 33]. To explore whether LASS2 suppresses tumour cell growth and metastasis through the ferroptosis signalling pathway, the CCK-8 assays, transwell migration and Matrigel invasion assays were performed on multiple tumour cell lines. We found that the co-treatment of LASS2 and Fer-1 or erastin still inhibited the viability of these tumour cells (BCPAP, MDA-MB-231, and Hepa1-6). However, the cell viability was higher in Adv-LASS2-GFP + Fer-1 group compared to the Fer-1 group, indicating the partially rescued effect of Fer-1 or erastin on LASS2 overexpressing tumour cells (Additional file 3: Fig. S2A–C). We confirmed that LASS2 overexpression significantly decreased the motility and invasiveness of multiple tumour cell lines (BCPAP, MDA-MB-231, Hepa1-6, and HepG2) compared to those of control cells (Fig. 6A and B). Interestingly, LASS2 overexpression also reversed the changes in migration and invasion induced by either Fer-1 or erastin in these tumour cell lines (Fig. 6A and B).

**Fig. 7:**
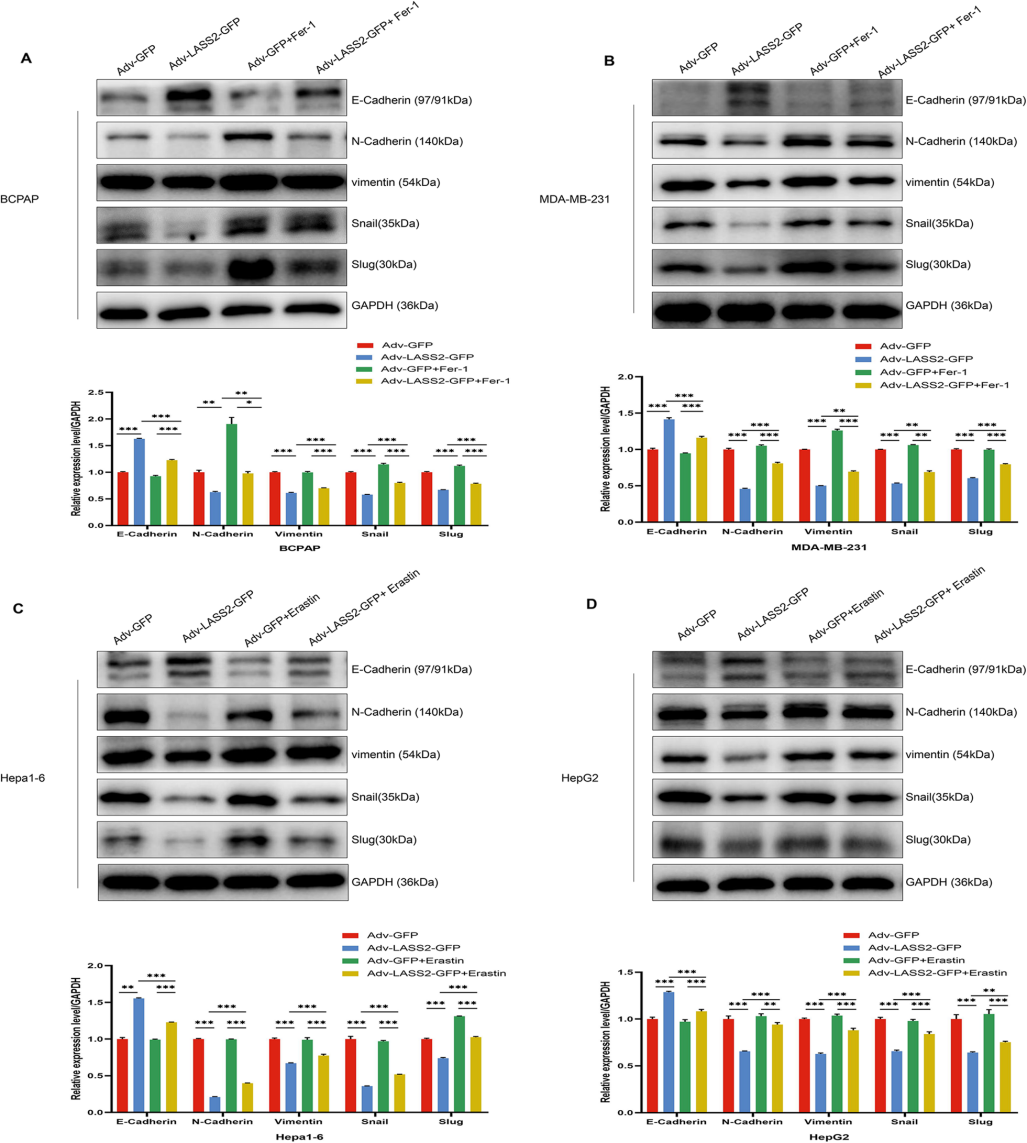
LASS2 overexpression affects EMT in tumour cell lines through ferroptosis. **A**–**D** Effects of LASS2 on the levels of E-Cadherin, N-Cadherin, vimentin, Snail, and Slug in various tumour cell lines detected by western blot. *P < 0.05, **P < 0.01, ***P < 0.001

It is well known that tumour cells can metastasize through epithelial-mesenchymal transition (EMT) [34]. As reported above, LASS2 inhibited tumour cell migration; therefore, we further investigated its role in EMT. Western blot analysis confirmed that LASS2 overexpression upregulated the expression of the EMT marker protein E-Cadherin and downregulated the expression of N-Cadherin, vimentin, Snail, and Slug, whereas the addition of ferroptosis agonists or inhibitors significantly rescued the effect of LASS2 (Fig. 7A–D) in the tumour cell lines. Collectively, our data suggest that LASS2 inhibits metastasis in multiple tumour cell lines, through the modulation of ferroptosis-associated mechanisms.

### Correlations between LASS2 and TFRC protein levels and iron content in multiple cancers

**Fig. 8:**
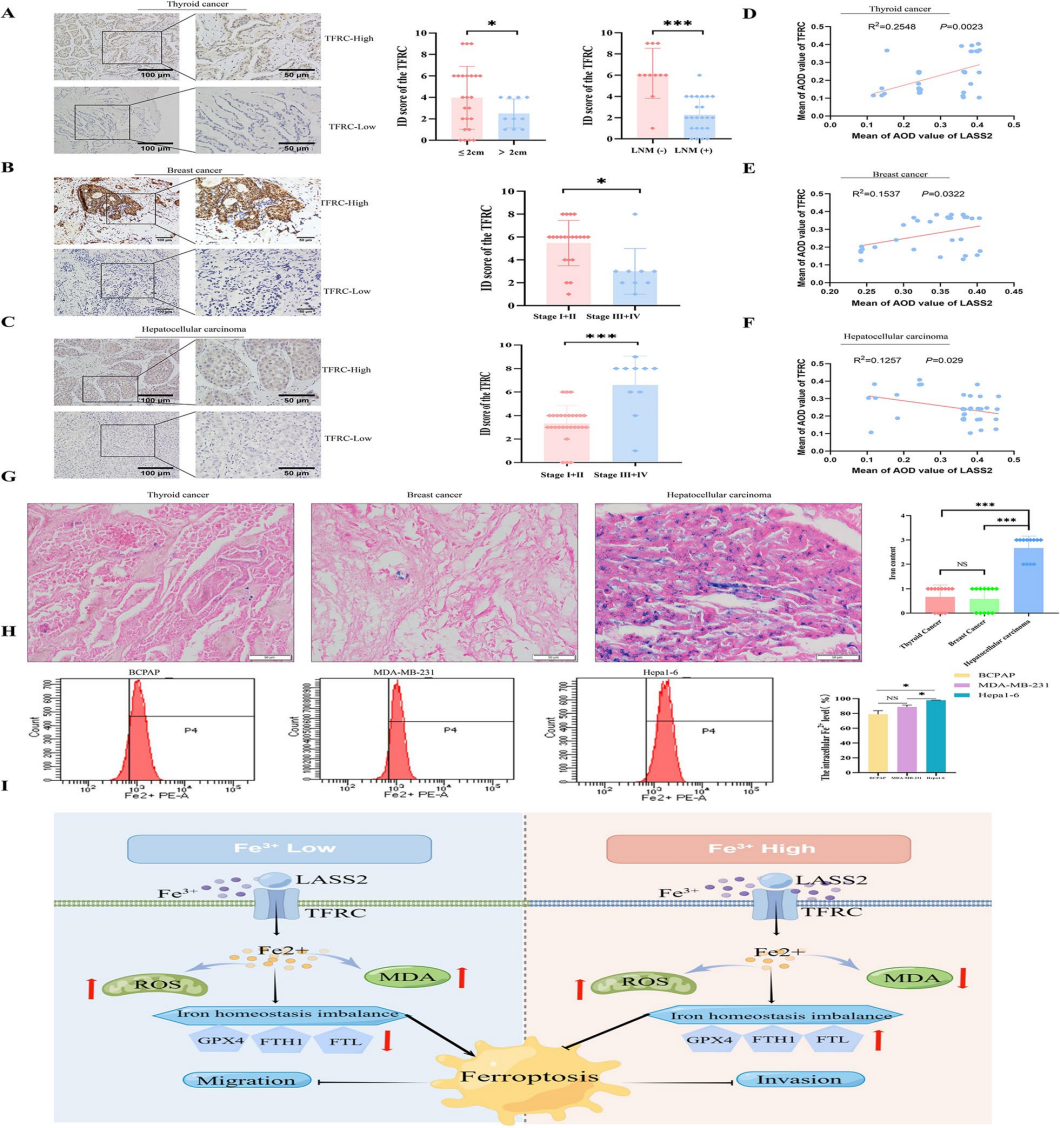
Levels of TFRC and iron content in tumour tissues. **A**–**C** The TFRC level in three tumour tissues and its correlation with clinicopathological parameters (scale bar: 100/50 μm). **D**–**F** Correlation analysis of the mean optical density values of LASS2 and TFRC. **G** Prussian blue staining for iron content in the three tumour tissues (scale bar: 50 μm). **H** Intracellular Fe^2+^ concentration was measured via flow cytometry. **I** Schematic diagram of the regulatory effect of LASS2 on the metastasis of multiple cancers via ferroptosis. *P < 0.05, ***P < 0.001

To assess the correlation between the levels of LASS2 and TFRC, we analysed 35 thyroid cancer, 30 breast cancer, and 38 HCC tumour tissues. The protein levels of TFRC in various cancer tissues were examined via IHC. We investigated the correlation between the TFRC protein level and clinicopathological characteristics in multiple patients with cancer (Table 2). In thyroid cancer, a low protein level of TFRC was correlated with tumour size (*P* = 0.017) and lymph node metastasis (*P* = 0.000) (Fig. 8A). Similarly, we found a negative correlation between the TFRC protein level and the clinical TNM stage in breast cancer (*P* = 0.015) (Fig. 8B). In contrast, the IHC results revealed that the TFRC protein level was positively correlated with the TNM clinical stage in HCC (*P* = 0.000) (Fig. 8C). Importantly, by analysing the average optical density values, we confirmed that the LASS2 protein level was positively correlated with the TFRC protein level in thyroid and breast cancer tissues (Fig. 8D and E) but negatively correlated with the TFRC protein level in HCC tissues (Fig. 8F). In addition, we analysed the ferric ion (Fe^3+^) content to assess whether there were differences in iron accumulation between multiple cancer tissues. As shown in Fig. 8G, using Prussian blue staining, iron accumulation was significantly greater in HCC tissue than in thyroid and breast cancer tissue. Furthermore, we compared the Fe^2+^ concentrations in the cells of these three tumorous using flow cytometry. Similarly, the results showed that the Fe^2+^ concentration in liver cancer cells was significantly greater than that in the other two cell types (Fig. 8H).

**Table 2:** Correlation between TFRC expression and clinicopathological parameters in three tumors

Characteristic	TFRC ID score										
	Thyroid cancer				Breast cancer				Hepatocellular carcinoma		
	0–4	6–12	*P* value		0–4	6–12	*P* value		0–4	6–12	*P* value
Sex			0.129	Age (years)			0.471	Sex			0.084
Male	10	7		< 51	9	9		Male	20	11	
Female	14	3		≥ 51	5	7		Female	7	0	
Age (years)			0.334	Tumor size			0.236	Age (years)			1.00
< 55	22	8		≤ 2 cm	2	3		< 50	9	3	
≥ 55	2	2		> 2 cm	12	13		≥ 50	18	8	
Tumor size			0.017*	TNM stage			0.015*	TNM stage			0.000*
≤ 2 cm	14	10		I	0	2		I	5	0	
> 2 cm	10	0		II	6	13		II	20	3	
LNM			0.000*	III	5	0		III	1	8	
Negative	2	9		IV	3	1		IV	1	0	
Positive	22	1		Metastases			0.249				
				Negative	3	1					
				Positive	11	15					
				LNM			0.183				
				Negative	11	9					
				Positive	3	7

## Discussion

Ferroptosis induction shows promise as a new anticancer treatment, but its regulation is complex. A comprehensive understanding of how ferroptosis proceeds in different tumour types is crucial for targeting individual cancers. Previous studies have focused on the function of LASS2 in individual cancer types [7, 35, 36], but this article is the first to explore the molecular mechanisms of LASS2 across multiple cancers.

In this study, we used bioinformatics analysis to examine variations in LASS2 expression in different cancer and paracancerous tissues, providing insight into the biological complexity of tumours. Following the analysis results, thyroid, breast, and liver cancer patients were chosen for initial validation due to their high incidence of malignant tumours with distant metastasis and their established association with ferroptosis. We found that low levels of LASS2 were associated with adverse clinical factors and poor prognosis in all three cancer types. Our functional studies also showed that LASS2 significantly inhibited the invasion and migration of these cancer cells, suggesting that this protein might act as a tumour suppressor. Further research will explore the role of LASS2 in other cancer types.

GSEA and our multiomics data showed that LASS2 overexpression led to enrichment of the ferroptosis signalling pathway in all three types of cancer cells, as evidenced by changes in morphology and oxidative stress indicators (ROS, MDA) that are characteristic of ferroptosis. Intriguingly, LASS2 exerted tumour-suppressive effects on all three types of cancer cells but had different effects on ferroptosis signalling.

Here, we determined that LASS2 interacts with TFRC in these specific cancer cell types. TFRC, also known as transmembrane glycoprotein transferrin receptor 1 (TFR1), is the predominant iron uptake protein on cell membranes; it regulates intracellular iron levels and plays a crucial role in maintaining iron homeostasis and ferroptosis [28, 37, 38]. Iron plays a pivotal role in cell viability and requires precise control of cellular iron homeostasis, including intracellular iron storage/release and import/export [39, 40]. Many cellular processes alter the sensitivity of cells to ferroptosis by altering the iron concentration, leading to cellular destabilization [39]. Ferritin heavy chain 1 (FTH1) and ferritin light chain (FTL) are the heavy and light chains of ferritin and are essential for iron storage. Our data showed that LASS2 overexpression promotes iron uptake by upregulating TFRC expression in thyroid and breast cancer cell lines while simultaneously reducing iron storage by downregulating FTL and FTH expression, thereby elevating labile iron, which further confers ferroptosis sensitivity. In contrast, LASS2 inhibited iron uptake and promoted iron storage in hepatoma Hepa1-6 and HepG2 cells, which in turn reduced their sensitivity to ferroptosis.

Furthermore, TFRC was found to regulate GPX4-dependent ferroptosis, and the overexpression of TFRC inhibits GPX4 [41]. Restoring GPX4 expression can suppress ferroptosis [42]. Our study showed that upregulation of LASS2 decreased GPX4 expression in thyroid and breast cancer cell lines but increased GPX4 expression in liver cancer cell lines, supporting the conclusions of Lu et al. [41]. Notably, overexpression of LASS2 reversed the changes in these cancer cells, such as changes in metastasis and ferroptosis-related protein levels (EMT, TFRC, GPX4, FTH1 and FTL1), induced by Fer-1 or erastin. Collectively, these data strongly demonstrated that LASS2 requires TFRC as a direct target to induce GPX4-dependent ferroptosis and thus inhibit tumour metastasis. Notably, the ferroptosis status caused by the overexpression of LASS2 in liver cancer cells was not consistent with that in thyroid and breast cancer cells. This discrepancy was attributed to the important role of the liver in systemic iron storage and homeostasis [43]. Here, we found that Fe^3+^ in HCC tumour tissues and Fe^2+^ in liver cancer cells were both significantly more abundant than in thyroid and breast tissue and tumour cells (there were no significant differences between thyroid and breast cancer tissue or cells). In addition, our co-IP LC/MS results showed different networks of interacting proteins. However, these findings still need to be further verified by subsequent studies and in various other cancer types.

## Conclusions

Our findings support the vital role of LASS2 in ferroptosis pathway signalling in cancer cell metastasis. Moreover, our study has revealed the previously unreported direct interaction between LASS2 and TFRC in various cancers. Mechanistic insights into the regulation of iron homeostasis by the LASS2-TFRC-GPX4 axis could lead to possible common therapeutic targets for cancer treatment.

### Supplementary Information

**Additional file 1: Table S1.** The sequence of primers for qPCR analysis.

**Additional file 2: Fig. S1.** An in vitro model of LASS2 overexpression was constructed

**Additional file 3: Fig. S2.** CCK-8 assay to analyse cell proliferation.

## Data Availability

Data are available on request from the authors.

## Abbreviations

LASS2: Longevity Assurance Homolog 2
GPX4: Glutathione peroxidase 4
TFRC: Transferrin receptor protein 1
ROS: Reactive oxygen species
OS: Overall survival
HCC: Hepatocellular carcinoma
IHC: Immunohistochemistry
ID: Intensity distribution
Fer-1: Ferrostatin-1
FTH1: Ferritin heavy chain 1
FTL: Ferritin light chain
PBS: Phosphate-buffered saline
MDA: Malondialdehyde
IP: Immunoprecipitation
LC–MS: Liquid chromatography–mass spectrometry
PLA: Proximity ligation assay
EMT: Epithelial-mesenchymal transition
