# Supplementary material for: LASS2 suppresses metastasis in multiple cancers by regulating the ferroptosis signalling pathway through interaction with TFRC
Source: Cancer Cell Int. 2024 Feb 28;24:87. doi: 10.1186/s12935-024-03275-8 (PMC10900749; doi:10.1186/s12935-024-03275-8)
Supplement: Supplementary file 1 — Additional file 1: Table S1. The sequence of primers for qPCR analysis. [file 12935_2024_3275_MOESM1_ESM.docx]

Table S1.

The sequence of primers for qPCR analysis

| Primer | Forward | Reverse |
| --- | --- | --- |
| *h*LASS2 | 5′-ATCGTCTTCGCCATTGTT-3′ | 5′-CGGTCACTGCGTTCATCT-3′ |
| *h*GAPDH | 5′-GGAGCGAGATCCCTCCAAAAT-3′ | 5′-GGCTGTTGTCATACTTCTCATGG-3′ |
| *m*LASS2 | 5′-CATCGTTTTCATCATCACTCG-3′ | 5′-TCACTGCGTTCATCTTCTATC-3′ |
| *m*GAPDH | 5′-GTGTTCCTACCCCCAATGTGT-3′ | 5′-ATTGTCATACCAGGAAATGAGCTT-3′ |
