# Supplementary material for: LASS2 suppresses metastasis in multiple cancers by regulating the ferroptosis signalling pathway through interaction with TFRC
Source: Cancer Cell Int. 2024 Feb 28;24:87. doi: 10.1186/s12935-024-03275-8 (PMC10900749; doi:10.1186/s12935-024-03275-8)
Supplement: Supplementary file 2 — Additional file 2: Fig. S1. An in vitro model of LASS2 overexpression was constructed [file 12935_2024_3275_MOESM2_ESM.docx]

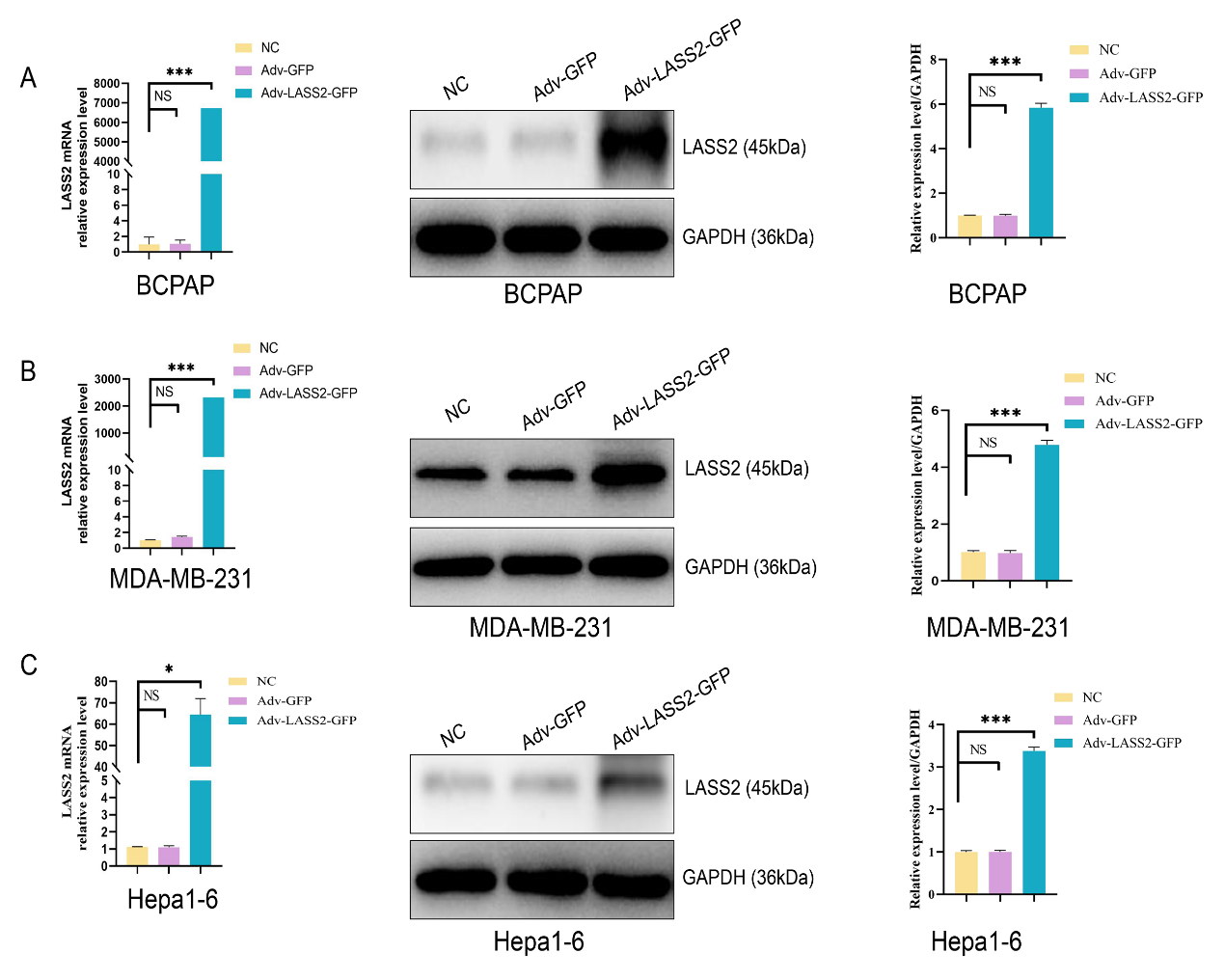


**Fig. S1** An in vitro model of LASS2 overexpression was constructed

in three tumour cell lines. **(A-C)** LASS2 overexpression at the mRNA and protein levels in BCPAP, MDA-MB-231, and Hepa1-6 cell lines was confirmed by RT‒qPCR and

Western blot.
