# Supplementary material for: LASS2 suppresses metastasis in multiple cancers by regulating the ferroptosis signalling pathway through interaction with TFRC
Source: Cancer Cell Int. 2024 Feb 28;24:87. doi: 10.1186/s12935-024-03275-8 (PMC10900749; doi:10.1186/s12935-024-03275-8)
Supplement: Supplementary file 3 — Additional file 3: Fig. S2. CCK-8 assay to analyse cell proliferation. [file 12935_2024_3275_MOESM3_ESM.docx]

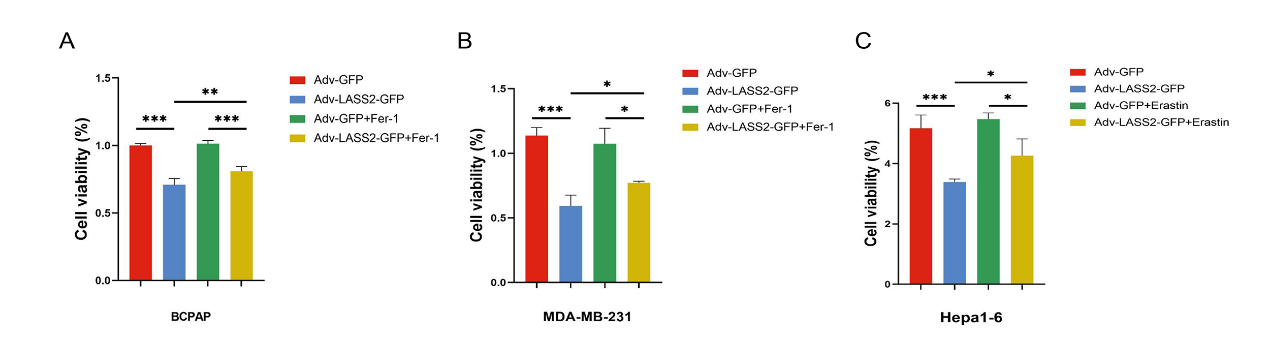


Fig. S2 CCK-8 assay to analyse cell proliferation. **(A-C)** Cell viability in each group was measured by the CCK-8 assay after BCPAP, MDA-MB-231, and Hepa1-6 cells were treated with Fer-1 or erastin or left untreated for 24 h and infected with Adv-*h*LASS2-GFP or Adv-*m*LASS2-GFP adenovirus for 48 h.
